# Production of a Highly Protease-Resistant Fungal α-Galactosidase in Transgenic Maize Seeds for Simplified Feed Processing

**DOI:** 10.1371/journal.pone.0129294

**Published:** 2015-06-08

**Authors:** Wenxia Yang, Yuhong Zhang, Xiaojin Zhou, Wei Zhang, Xiaolu Xu, Rumei Chen, Qingchang Meng, Jianhua Yuan, Peilong Yang, Bin Yao

**Affiliations:** 1 Feed Research Institute, Chinese Academy of Agricultural Sciences, Beijing, P. R. China; 2 Biotechnology Institute, Chinese Academy of Agricultural Sciences, Beijing, P. R. China; 3 Institute of Food Crops, Jiangsu Academy of Agricultural Sciences, Nanjing, P. R. China; Institute of Genetics and Developmental Biology, Chinese Academy of Sciences, CHINA

## Abstract

Raffinose-family oligosaccharide (RFO) in soybeans is one of the major anti-nutritional factors for poultry and livestocks. α-Galactosidase is commonly supplemented into the animal feed to hydrolyze α-1,6-galactosidic bonds on the RFOs. To simplify the feed processing, a protease-resistant α-galactosidase encoding gene from *Gibberella* sp. strain F75, *aga-F75*, was modified by codon optimization and heterologously expressed in the embryos of transgentic maize driven by the embryo-specific promoter ZM-leg1A. The progenies were produced by backcrossing with the commercial inbred variety Zheng58. PCR, southern blot and western blot analysis confirmed the stable integration and tissue specific expression of the modified gene, *aga-F75m*, in seeds over four generations. The expression level of Aga-F75M reached up to 10,000 units per kilogram of maize seeds. In comparison with its counterpart produced in *Pichia pastoris* strain GS115, maize seed-derived Aga-F75M showed a lower temperature optimum (50°C) and lower stability over alkaline pH range, but better thermal stability at 60°C to 70°C and resistance to feed pelleting inactivation (80°C). This is the first report of producing α-galactosidase in transgenic plant. The study offers an effective and economic approach for direct utilization of α-galactosidase-producing maize without any purification or supplementation procedures in the feed processing.

## Introduction

Soybean meal is used extensively in animal feed as a rich source of proteins, but it contains many anti-nutritional factors that cause gastrointestinal disorders [1]. One of them is raffinose family oligosaccharide (RFO; mainly raffinose and stachyose) that is composed of d-galactopyranose units attached to sucrose through α-1,6-glycosidic bond [2]. RFO can cause flatulence, nausea and discomfort [3], and may increase the viscosity of digesta, thereby decrease the interaction with digestive enzymes (such as trypsin) and interfere with nutrient digestion [4]. As a result, it negatively affects energy or protein digestibility and growth of livestock. α-d-Galactoside is hard to be degraded in the small intestine because humans and monogastric animals lack α-galactosidase [5]. It has been proved that the nutritive value of soy products treated by α-galactosidase alone or in combination with other enzymes is significantly improved for animal consumption [6–8]. Thus it’s a common practice to supplement α-galactosidase into feed to alleviate the anti-nutritional effects of RFOs.

α-Galactosidase (α-d-galactoside galactohydrolase; EC3.2.1.22) or melibiase is an exo-type glycoside hydrolase (GH) that catalyzes the removal of terminal α-1,6-linked nonreducing galactose residues from different substrates [9]. It has been classified into GH families 4, 27, 36, 57, 97 and 110 in the CAZy database (http://www.cazy.org/Glycoside-Hydrolases.html) [10]. α-Galactosidases are widely distributed in plants, mammals and microorganisms [11–14], and many of them have been extensively studied with regard to potential applications in various industries. The application of α-galactosidase to hydrolyze α-d-galactoside prior to ingestion would allow legumes to be used as a sugar supplement in foods with low sugar content [15]. Although the biochemical properties and catalytic mechanism of α-galactosidases are well known, how to use it effectively in feed industry still needs further studies. Currently, the production of commercial α-galactosidase by microbial expression systems (i.e. *Escherichia coli* and *Pichia pastoris*) [8,16] has some disadvantages like high equipment cost, high energy consumption, serious environment pollution, complex processing, etc. An efficient and economic alternative is to produce α-galactosidase directly in feed grains as β-mannanase and xylanase produced in transgenic maize [17] and rice [18], respectively.

Transgenic plants have been developed through different genetic engineering techniques and appear to play an important role in environmental protection and global food supplies. In 2011, transgenic plants were produced in 29 countries, and transgenic maize accounted for nearly one third of the total genetically modified crops [19]. Maize as the main ingredient of animal feed (nearly 50%) is an ideal natural bioreactor in which a β-glucanase gene from *Bispora* sp. MEY-1 has been successfully expressed with the activity of 779,800 U/kg seeds [20]. The development of transgenic maize will not only reduce the loss of resources and simplify the production process, but also provide an environmentally friendly approach to produce feed enzymes.

In this study, we first developed a genetically stable maize line that shows α-galactosidase activity only in seeds. The α-galactosidase of GH 36, Aga-F75, from *Gibberella* sp. strain F75 [16] was selected for its excellent enzymatic properties, such as acidic pH optimum (pH 4.0), broad pH stability (pH 3.5–10.0), broad substrate specificity and strong protease resistance. Moreover, it had positive synergic effect with trypsin, which activity was promoted in the presence of trypsin. The gene codon was optimized for better expression in maize. Its genetic stability and enzyme properties were also assessed and compared with its counterpart produced in *P*. *pastoris*.

## Materials and Methods

### Materials

Maize Hi-II [21] was selected as the host variety for α-galactosidase production. N6 1-100-25 medium [22] containing 0.2% (w/v) phytagel (Sigma, St. Louis, MO, USA) was used to preserve the immature embryos for callus induction. The commercial maize inbred-line Zheng58 was used to produce progenies by hybridizing with transgenic maize for its genetic stability.

The substrate 4-nitrophenyl α-D-galactopyranoside (*p*NPG) was purchased from Sigma. All the restriction endonucleases were purchased from New England Biolabs (Ipswich, MA, USA), and the T4 DNA ligase was purchased from Promega (Madison, WI, USA).

### Production of recombinant Aga-F75 in *P*. *pastoris*

Preparation of all media and protocols for heterologous expression of *aga-F75* in *P*. *pastoris* GS115 followed the *Pichia* expression manual (Invitrogen, Carlsbad, CA, USA). The gene fragment coding for mature Aga-F75 without the signal peptide-coding sequence was cloned into vector pPIC9 to construct the recombinant plasmid pPIC9-*aga-F75*, which was further linearized and transformed into *P*. *pastoris* GS115 competent cells by electroporation. Positive transformants were screened based on their α-galactosidase activities as described below. The transformant showing the highest activity was selected for large-scale fermentation in 1-l conical flasks. To purify recombinant Aga-F75, the crude enzyme was sequentially loaded onto the HiTrapTM Desalting column and HiTrap Q Sepharose XL FPLC column from GE Healthcare (Uppsala, Sweden). The purity of Aga-F75 was identified to be 90% based on SDS-PAGE. Purified Aga-F75 was deglycosylated with PNGase F following the instruction of manufacturer (New England Biolabs, Ipswich, MA, USA).

### Modification of the α-galactosidase gene *aga-F75*

In order to enhance the expression efficiency in transgenic maize, the DNA sequence of *aga-F75* from *Gibberella* sp. F75 (FJ392036) [16] was modified by removing the putative N-terminal signal peptide-coding sequence, introducing two restriction sites (*Bam*HI and *Avr*II) at both ends of the gene and optimizing the codons according to the optimal codon usage and known codon bias of maize [23,24]. Codon adaptation index (CAI) and GC content were analyzed by using the GenScript Rare Codon Analysis Tool (http://www.genscript.com/cgi-bin/tools/rare_codon_analysis) to assess the modified gene coding sequence and predict the gene expression level. The modified gene, designated *aga-F75m*, was synthesized by Genscript (Nanjing, China), and cloned into vector pUC57MCS to construct the recombinant plasmid pUC57-*aga-F75m*.

### Plasmid construction

**Fig 1:**
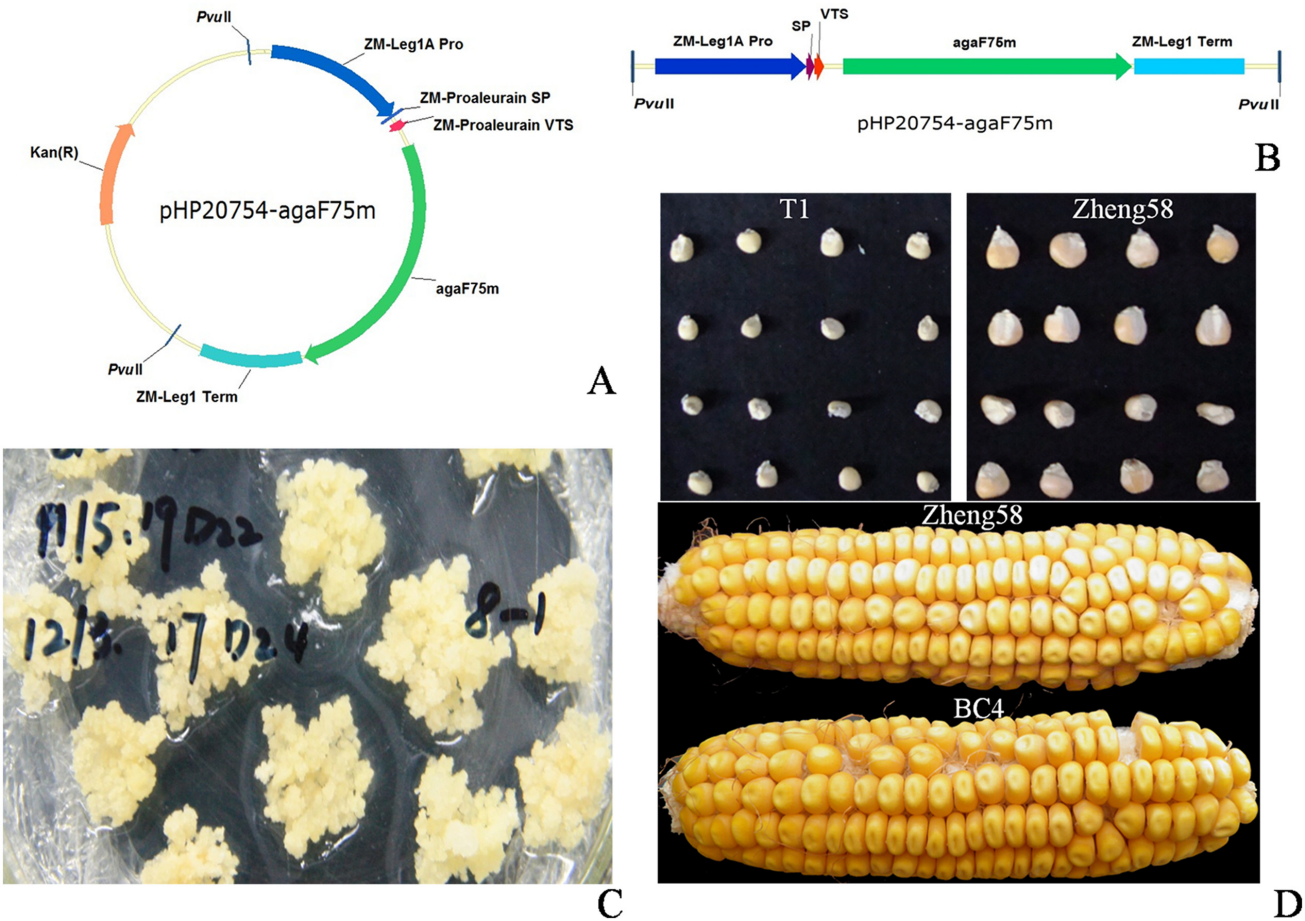
Construction of the recombinant vector and transformation into maize seeds. (A) The recombinant expression vector pHP20754-*aga-F75m*. (B) The chimeric gene cassette for expression in maize. (C) Embryogenic calli in selective medium. (D) Transgenic maize seeds of generations T1 and ear of BC4 in comparison with that of wide-type Zheng58.

The expression vector pHP20754 consists of the ZM-leg1A promoter that is endosperm specific, the vacuole targeting sequence (VTS), the ZM-leg1 terminator and the maize proaleurain signal peptide. The region between the promoter and terminator is the target of introduction of *aga-F75m* (Fig 1A). The plasmid pUC57-*aga-F75m* and vector pHP20754 were both digested with *Bam*HI and *Avr*II and ligated by T4 DNA ligase to construct the expression plasmid pHP20754-*aga-F75m*. For transformation in maize Hi-II, the expression plasmid was digested with *Pvu*II to produce the chimeric gene expression cassette (Fig 1B).

The plasmid pHP17042BAR was used as the selectable marker for transformation. It contains the maize histone H2B promoter, the maize Ubiquitin 5'-UTR intron-1, the potato protease II terminator [25] and the *bar* gene, which was excised from pHP17042BAR by *Hind*III, *Xho*I and *Sac*I. The *bar* gene from *Streptomyces hygroscopicus* was used to screen the positive transgenic plants.

### Transformation, selection and regeneration of maize

The chimeric gene expression cassette and the plasmid fragment containing the *bar* gene were mixed equivalently and adjusted to 200 ng/μl. Transformation into maize Hi-II cells was carried out with high-velocity microprojectiles (Bio-Rad, Hercules, CA, USA) wrapped by the aim DNAs [26,27]. After recovery, the embryonic calli was transferred onto the selective medium supplemented with bialaphos (the marker for selection). The positively transformed calli were cultivated in differentiation medium and rooting medium [28] in succession, and the seedlings (T0 plants) were transplanted into greenhouse. Inbred-line Zheng58 was used as the male parent to produce T1 seeds by being pollinated with T0 plants. The BC1 to BC4 generations were also produced by the backcross method.

### PCR detection of exogenous gene

The genomic DNA was isolated from the transgenic maize leaves by CTAB method [29]. The specific primers F75m-F (5′-CGTTGAGCTGGACCCATCGGATC-3′) and 20754-398R (5′-TTCCTGGCAAATCACTCGGTGTATC-3′) were used to verify the inheritance of *aga-F75m* in maize. The genomic DNA of Zheng58 and the recombinant plasmid pHP20754-*aga-F75m* were used as the negative and positive controls, respectively. The *actin* gene was amplified by primers AC326F (5′-ATGTTTCCTGGGATTGCCGAT-3′) and AC326R (5′-GCATCACAAGCCAGTTTAACC-3′) and used as the control to check the quality of genomic DNA.

### Southern blot

Five grams of transgenic maize leaves were ground into a fine powder in liquid nitrogen and subject to DNA extraction as described above. Negative control was the genomic DNA of Zheng58. Genomic DNA (~50 μg) was digested by *Hind*III and *Bam*HI, separated on a 0.8% (w/v) agarose gel and transferred onto a hybond-N+ nylon membrane (GE Healthcare, Uppsala, Sweden) with a Trans-Blot SD system by UV-crosslinking. A 600 bp fragment of *aga-F75m* was labeled by digoxin and used for in-situ hybridization as the probe following the instructions of DIG-High prime DNA labeling and detection starter kit II (Roche, Indianapolis, IN, USA).

### Western blot

Dried maize seeds of the transgenic lines and Zheng58 were smashed into a fine powder, respectively, with a high-throughput tissue homogenizer Geno/Grinder 2010 (SEPX CertiPrep, Metuchen, NJ, USA). The seed powder of each sample, approximately 30 mg, was put into a 1.5 ml tube containing 300 μl of 50 mM citric acid-Na_2_HPO_4_, pH 4.0 (extraction buffer), and agitated on a shaker for 1 h at room temperature. After centrifugation at 5,000 × *g* for 10 min, the supernatant of seed extract was collected and incubated in pro-cooled acetone at the ratio of 1:2 for 30 min. The mixture was centrifuged at 14,000 × *g* for 15 min, and the supernatant was removed. The seed protein was dissolved in 30 μl of deionized water, and was equally divided into two parts. One part was deglycosylated with PNGase F as described above, and the other was maintained at −20°C for other analysis.

Protein extracts of maize Zheng58 and purified Aga-F75 from *P*. *pastoris* were used as the negative and positive controls, respectively. Proteins from the root, stem and leaf of a transgenic plant of generation BC1 were extracted as described above and used for tissue specificity analysis.

The preparation of polyclonal antibody of Aga-F75 in rabbits (6 pounds and greater) was conducted in the Laboratory of Animal Center, Institute of Genetics and Developmental Biology, Chinese Academy of Sciences (Beijing, China) following the rules and protocols approved by the Experimental Animal Ethics Committee of Chinese Academy of Sciences. The purification of polyclonal antibody and the procedures of Western blot were carried out as described previously [17]. The bands were then excised from the gel for protein identification by matrix assisted laser desorption/ionization time of flight (MALDI-TOF) mass spectrometry at Tianjin Biochip Corporation (Tianjin, China).

### α-Galactosidase activity assay and enzyme characterization

Crude maize proteins were extracted from five randomly selected seeds as described above, and the supernatant was used for α-galactosidase activity assay. The α-galactosidase activity was measured by using a modified *p*NPG method [15,30]. One unit of α-galactosidase activity was defined as the amount of enzyme that released 1 μmol of *p*NP per minute at the assay conditions (pH 4.0 and 60°C). The reaction system including 50 μl of enzyme sample, 200 μl of 100 mM McIlvaine buffer, and 250 μl of substrate solution (2 mM *p*NPG in 100 mM McIlvaine buffer) was incubated at 60°C for 10 min, followed by addition of 1.5 ml of 1 M Na_2_CO_3_. The absorption was measured at 405 nm. All α-galactosidase activity assays were performed in triplicate.

The crude extract of BC2 seeds was used for enzyme characterization. The optimal pH for the α-galactosidase activity of Aga-F75M was determined at 37°C for 10 min in buffers of 100 mM McIlvaine buffer (pH 2.0–8.0), 100 mM Tris-HCl (pH 8.0–9.0) and 100 mM glycine-NaOH (pH 9.0–10.0). The stability of Aga-F75M under different pH conditions was assessed by measuring the residual activities under standard conditions after incubation of the enzyme in the buffer as described above at 37°C for 1 h. The optimal temperature for Aga-F75M activity was carried out at various temperatures from 30 to 80°C at the experimentally determined optimal pH. Thermal stability was determined by measuring the residual activities under standard conditions after incubating the enzyme at 60°C and 70°C for different durations without substrate.

The protease-resistance of Aga-F75M was also determined. The seed extract was prepared by incubating 100 mg of maize seed powder at room temperature in 1 ml of distilled water by 1-h agitation on a shaker and centrifugation at 12,000 *g* for 10 min. Four proteases, including trypsin, α-chymotrypsin, proteinase K and subtilisin A, were dissolved in 100 mM citric acid-Na_2_HPO_4_, pH 7.0 or pH 7.5 to the concentration of 100 mg/ml. The seed extract and each protease were mixed at 1:10 ratio (v/v), and the mixtures were incubated at 37°C for 30 min and 60 min followed by enzyme activity assay. Blank controls without any protease were treated under the same conditions.

### Anti-activation stability over feed pelleting process

Feed pelleting was carried out as descripted previously [17,20]. α-Galactosidase activities and dry matter contents (DM) were measured before and after the pelleting process. Equal amounts of crude Aga-F75 based on α-galactosidase activity (337 U/kg) were added into Zheng58 seeds, followed by same treatments. The percentages of α-galactosidase activity lost were determined by comparing the activities before and after pelleting.

## Results

### Codon optimization and construction of embryo-specific vector pHP20754-*aga-F75m*

By using codon optimization and gene modification, the CAI value and GC content of *aga-F75m* were increased from 0.71 to 0.82 and 51.29% to 55.82%, respectively. These higher values are better for exogenous gene expression in maize. As a result, *aga-F75m* codes for the same polypeptide as *aga-F75* but only shares74.9% nucleotide sequence identity with *aga-F75*.

*aga-F75m* was first subcloned into vector pUC57, which was further digested and ligated with vector pHP20754 to construct the expression plasmid pHP20754-*aga-F75m*. pHP20754-*aga-F75m* was then transformed into the calli of maize Hi-II regenerated on bialaphos medium (Fig 1C) and verified by PCR analysis.

### Plant regeneration and phenotypic evaluation

The regenerated young plants showed good growth in the greenhouse. A total of 33 independent transgenic events were obtained, and 619 T1 seeds of four events 8–1, 11–1, 11–2 and 14–2 were selected to grow in fields and backcrossed with Zheng58 to produce progenies. As shown in Fig 1D, T1 transgenic plants and Zheng58 showed different seed phenotypes, but the maize ear of generation BC4 having 93.75% genetic resources of Zheng58 had no visible morphological changes from Zheng58.

### Determination of exogenous gene integration

**Fig 2:**
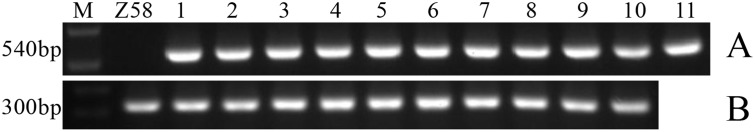
PCR analysis of *aga-F75m* (A) and *actin* (B) in the transgenic maize leaves of transformation event 8–1. Lanes: M, the DNA molecular weight markers; Z58, the non-transgenic Zheng58; 1–10, the transgenic plants; 11, the *aga-F75* as the positive control.

PCR reactions with primers specific for *aga-F75m* were conducted to identify the positive transgenic plants of generations T1 to BC4. A 539 bp gene fragment was detected in the transformation events 8–1 (Fig 2A) and 11–2. The bright band of *actin* gene (~300 bp) indicated the successful extraction of genomic DNA (Fig 2B). According to the PCR results, transgenic plants showed excellent stability in genetic inheritance until BC4.

**Fig 3:**
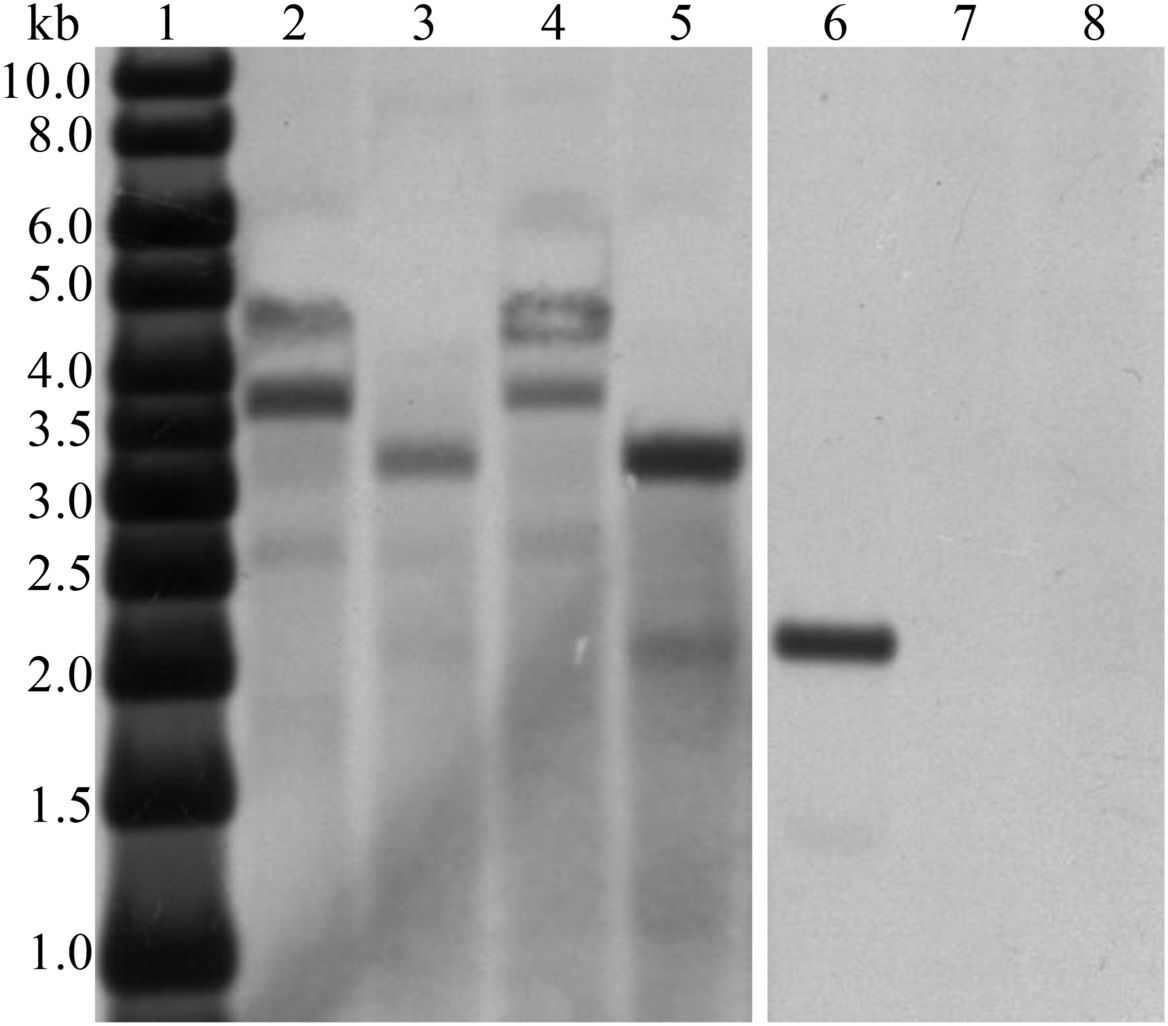
Southern blot analysis of *aga-F75m* in two transgenic plants. Lanes: 1, the DIG-labeled molecular weight markers; 2 and 4, the *aga-F75m* fragment with *Bam*HI digest; 3 and 5, the *aga-F75m* fragment with *Hind*III digest; 6, the digested expression cassettes as a positive control; 7 and 8, the genome of non-transgenic Zheng58 digested by *Bam*HI and *Hind*III, respectively.

The genomic DNAs of two positive transgenic plants of event 8–1 were analyzed by southern blot after restriction digest with *Bam*HI and *Hind*III (Fig 3). After *Bam*HI digest, two bands of ~3.7 and ~4.8 kb were detected in the lanes of transgenic plants, but not in non-transgenic Zheng58. There is no *Hind*III site in the *aga-F75m*. When the gene expression cassette was treated twice with *Hind*III, an internal fragment of ~3.4 kb was released. These results indicated that there are at least two copies of *aga-F75m* in the maize genome.

### Evaluation of gene expression

**Fig 4:**
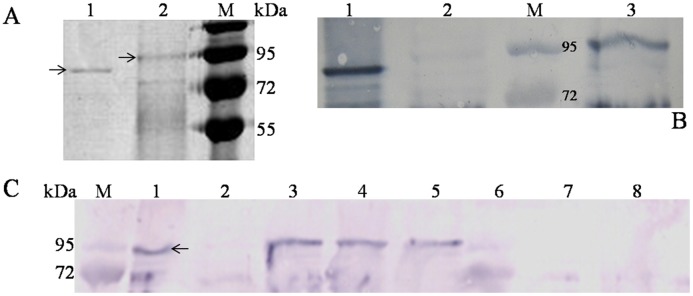
SDS-PAGE and western blot analysis of gene expression in *P*. *pastoris* and transgenic maize. (A) SDS-PAGE analysis of recombinant Aga-F75 produced in *P*. *pastoris*. Lanes: 1, the purified Aga-F75 with PNGase F treatment; 2, the purified Aga-F75; M, the protein molecular markers. (B) Western blot analysis of Aga-F75M produced in transgenic maize. Lanes: 1, the protein extract of transgenic maize seeds with PNGase F treatment; 2, the protein extract of non-transgenic Zheng58 seeds as a negative control; M, the protein molecular markers; 3, the protein extract of transgenic maize seeds. (C) Specific expression of Aga-F75M in transgenic maize. Lanes: M, the protein molecular markers; 1, the purified recombinant Aga-F75 produced in *P*. *pastoris*; 2, the protein extract of non-transgenic Zheng58 seeds; 3–5, the protein extracts of transgenic maize seeds; 6–8, the protein extracts of leaf, stem, and root of the transgenic plant, respectively.

The positive control, recombinant Aga-F75 produced in *P*. *pastoris*, showed a band of 95 kDa on SDS-PAGE, much higher than its theoretical molecular weight (~82 kDa). After PNGase F treatment, there was a reduction in molecular weight (Fig 4A). The result indicated that *N*-glycosylation occurred in Aga-F75 during its heterologous expression in *P*. *pastoris*.

**Fig 5:**
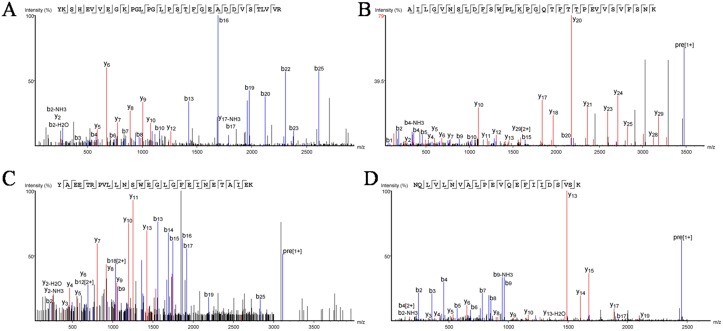
Peptides of Aga-F75M identified by MALDI-TOF mass spectrometry.

Crude proteins were extracted from dried maize seeds of the transgenic event 8–1 for the assessment of heterologous expression efficiency of *aga-F75m*. Western blot analysis showed that no band was detected in the negative control (Zheng58). The recombinant Aga-F75M from transgenic maize showed one main band of approximately 95 kDa on the PVDF membrane after hybridization with the antibody (Fig 4B). Further PNGase F treatment reduced the molecular weight of Aga-F75M, suggesting the occurrence of *N*-glycosylation in Aga-F75M when expressed in transgenic maize. Moreover, proteins extracted from the root, stem and leaf of the positive lines showed no objective band (Fig 4C), indicating the expression of Aga-F75M was tissue specific under the control of endosperm specific ZM-leg1A promoter. MALDI-TOF analysis of the band verified the identity of Aga-F75M (Fig 5).

### Evaluation of seed-derived α-galactosidase activity

Positive transgenic maize seeds of event 8–1 were selected for α-galactosidase activity assays. The maximal α-galactosidase activity of BC4 seeds reached up to 10,000 U/kg. Of all tested positive plants, the percentage of seeds with α-galactosidase activities of more than 1,000 U/kg was about 80%. The results indicated that the heredity of *aga-F75m* is stable in maize until BC4.

**Fig 6:**
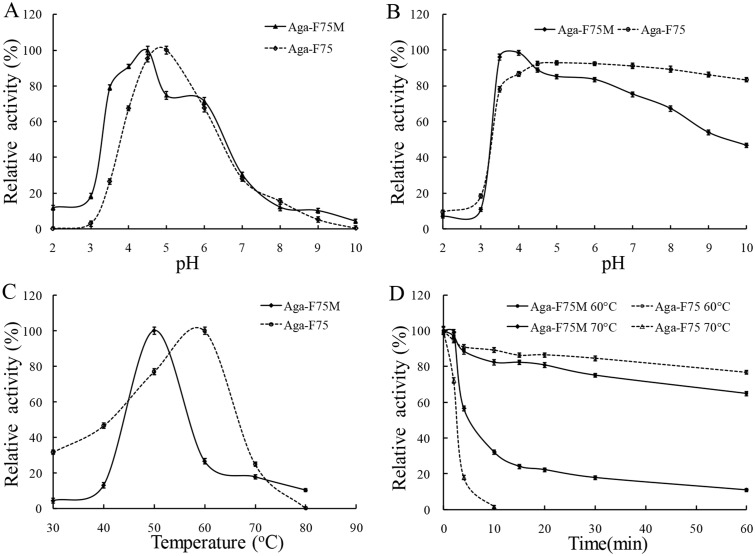
Enzyme properties of recombinant Aga-F75M produced in transgenic maize. (A) Effect of pH on Aga-F75M activity. (B) pH stability of Aga-F75M. (C) Effect of temperature on Aga-F75M activity. (D) Thermostability of Aga-F75M pre-incubated at 60°C or 70°C.

The crude proteins of transgenic maize seeds were characterized and compared with the spray dried fermentation broth of *P*. *pastoris*-derived Aga-F75. Aga-F75M showed an optimal pH (4.5) similar to Aga-F75 (pH 5) (Fig 6A), but exhibited worse stability over alkaline pH range. Aga-F75M retained more than 50% of the initial activity after incubation at pH 3.5 to 10.0, 37°C for 1 h (Fig 6B). The temperature optimum of Aga-F75M was 50°C, 10°C lower than that of Aga-F75 (Fig 6C). Aga-F75M remained more activity after incubation at 70°C for 60 min (Fig 6D). Aga-F75M was highly resistant to most neutral proteases tested. After 60 min incubation, Aga-F75M retained 91%, 89%, and 70% of the initial activities in the presence of trypsin, α-chymotrypsin and subtilisin A, respectively. When incubated with proteinase K for 30 min, the enzyme only retained 20% activity.

### Evaluation of the anti-inactivation stability over feed pelleting

The α-galactosidase activities of Aga-F75M and Aga-F75 were set to 337 U/kg. After pelleting at 80°C, 100°C or 120°C, Aga-F75 lost more activities than Aga-F75M, indicating that Aga-F75M was more stable over pelleting process than Aga-F75 (Table 1).

**Table 1:** Thermostability of Aga-F75 and Aga-F75M during feed pelleting ^a^.

	α-Galactosidase activity (U/kg)		
Enzymes	Before pelleting	After pelleting	Lost activity (%)
**Aga-F75**			
80°C	337	1	99
100°C	337	0	100
120°C	337	0	100
**Aga-F75M**			
80°C	337	59	82
100°C	337	32	90
120°C	337	24	93

^a^ Aga-F75 and Aga-F75M were the recombinant proteins produced in *P*. *pastoris* and transgenic maize seeds, respectively.

## Discussion

There is a consistent increase in the use of genetically modified plants for food or feed. However, most studies have been focusing on plant resistance against disease [31] and insect [32] rather than on the improvement of nutrition utilization. Enzyme plays an important role in the feed industry, and its direct production in feed grains without extra industrial process would be more convenient and economic for application in feed [17]. Maize is an important food and feed crop around the world. The first transgenic Bt maize was produced in the mid-1990s, since then, maize has become one of the most important target crops for biotechnological innovation. Currently, there are more biotech traits available on the market in maize than in any other crop [33]. In this study, we also selected maize for the production of a fungal α-galactosidase of GH 36.

To our knowledge, this is the first report of expression of an α-galactosidase in forage crop. α-Galactosidases play an important role in both feed industry and medical industry. Up to now, there are many α-galactosidase transgenic animals, which are used to solve medical problems. For example, a double transgenic pig with α-1,2-fucosyltransferase and α-galactosidase was designed to avoid hyperacute xenograft rejection [34]. But α-galactosidase as an essential feed enzyme has not been transferred into feed grains yet. In the study, we developed an α-galactosidase transgenic maize to simplify the process of feed production. Producing α-galactosidase in transgenic maize directly without any industrial processing requires less energy and resources than microbial expression systems.

An excellent transformation receptor maize Hi-II, which is the most commonly used because of its reliable and high transformation efficiency in laboratories [33], and a strong embryo-specific promoter ZM-leg1A were used to improve the transformation efficiency and propagation from transgenic lines with high enzyme activities [22,25]. As a result, the average and maximum α-galactosidase activities in transgenic maize seeds were up to 3,500 U/kg and 10,000 U/kg of seeds, respectively, which are high enough for direct application in feed. However, compared to other MAN5AS [17] and Bgl7AM [20] produced in plants, Aga-F75M showed a lower expression level in seeds. The reason might be that the molecular weight of Aga-F75M (~95 kDa) is much higher than that of other plant-derived proteins (<60 kDa). No α-galactosidase activity was detected in the root, stem and leaf of a positive line, and western blot analysis proved the endosperm-specific expression of Aga-F75M. The protein band detected by western blot analysis showed that Aga-F75M had a higher molecular weight (~95 kDa) than the calculated value (~82 kDa) and showed reduction after PNGase F treatment. It suggested that Aga-F75M was successfully expressed in maize seeds with *N*-glycosylation.

Transgenic lines of *aga-F75m* showed normal phenotype as wild-type Zheng58. It might be ascribed to the specific expression of *aga-F75m* in maize seeds instead of other tissues, which lessened the potential impairment to plants. But the effects of *aga-F75m* insertion on the maize seeds need to be determined in future study. Aga-F75M showed more activity than that of Aga-F75 in acid pH (pH 2.0–4.5) with the peak activity at pH 4.5, which is consistent with the physiological conditions of chicken stomach (pH 2.8–4.8) [35]. Moreover, Aga-F75M was biologically active in a broad pH range and exhibited higher acidic stability, and remained more activity after incubation at 70°C for 60 min and after pelleting at 80°C. These data indicated that Aga-F75M would be stable within the acid environment and exhibit greater application in feed industry than Aga-F75.

In conclusion, we successfully constructed a tissue-specific vector for expressing the α-galactosidase gene in transgenic maize seeds. It’s the first time to produce recombinant α-galactosidase directly in forage crop on a large scale. The enzyme had similar properties as its counterpart produced in *P*. *pastoris* but showed better anti-activation stability against feed pelleting. The development of transgenic maize will provide an environment-friendly and low-cost approach to produce feed enzymes with social and ecological significance. In future studies its application effectiveness will be evaluated in the feed industry.
